# Use of antidepressants and the risk of myocardial infarction in middle-aged and older adults: a matched case-control study

**DOI:** 10.1007/s00228-015-1972-2

**Published:** 2015-11-07

**Authors:** Raymond Noordam, Nikkie Aarts, Maarten J. G. Leening, Henning Tiemeier, Oscar H. Franco, Albert Hofman, Bruno H. Stricker, Loes E. Visser

**Affiliations:** Department of Internal Medicine, Erasmus MC-University Medical Center Rotterdam, Rotterdam, The Netherlands; Department of Epidemiology, Erasmus MC-University Medical Center Rotterdam, P.O. Box 2040, 3000 CA Rotterdam, The Netherlands; Department of Cardiology, Erasmus MC-University Medical Center Rotterdam, Rotterdam, The Netherlands; Inspectorate of Health Care, Utrecht, The Netherlands; Apotheek Haagse Ziekenhuizen-HAGA, The Hague, The Netherlands

**Keywords:** Antidepressive agents, Case-control studies, Myocardial infarction, Serotonin, Selective serotonin reuptake inhibitors

## Abstract

**Purpose:**

Antidepressants, specifically selective serotonin reuptake-inhibiting antidepressants (SSRIs), decrease platelet activation and aggregation in in vitro experiments and could therefore decrease the risk of myocardial infarction (MI). However, prior studies addressing this hypothesis showed contradictory results. Our purpose was to investigate the association between the use of any antidepressant drug and incident MI among middle-aged and older adults.

**Methods:**

We embedded a case-control study in the prospective Rotterdam Study (1991–2011). Controls were matched to MI cases based on sex and age at the same calendar date, and confounding factors were taken into account as time-varying covariates. The relative risk of MI during current and past use of an antidepressant was analyzed with conditional logistic regression with never use of antidepressant drugs as the reference category.

**Results:**

A total of 744 out of a cohort of 9499 study participants developed MI during follow-up. After statistical adjustment for traditional cardiovascular risk factors and depression, current use of any antidepressant was associated with a lower risk of MI (odds ratio (OR), 0.71; 95 % confidence interval (CI), 0.51–0.98) compared with never use of any antidepressant. SSRI use showed the lowest relative risk (OR, 0.65; 95 % CI, 0.41–1.02), albeit marginally not statistically significant. Past use of any of the antidepressant classes was not associated with a lower risk of MI.

**Conclusions:**

Current use of antidepressants was associated with a lower risk of MI. Of the different classes, the use of SSRIs showed the lowest risk of MI, and therefore confirming the research hypothesis.

**Electronic supplementary material:**

The online version of this article (doi:10.1007/s00228-015-1972-2) contains supplementary material, which is available to authorized users.

## Introduction

Findings from in vitro experiments have led to the hypothesis that the use of antidepressant drugs, and specifically use of selective serotonin reuptake inhibitors (SSRIs), might decrease the risk of myocardial infarction (MI). The inhibition of the serotonin reuptake transporter on blood platelets by SSRIs decreases platelet activation and aggregation in in vitro experiments [1, 2]. Similarly, antidepressants other than SSRIs might decrease platelet activation and aggregation by antagonizing the serotonin receptor and/or inhibiting also serotonin reuptake, although with lower affinity than SSRIs [3, 4].

Studies addressing the hypothesis of a lower risk of MI during use of SSRIs showed inconsistent results. Some studies reported a lower risk of MI in users of SSRIs [5, 6], while others showed no decreased risk [7–10] or an increased risk [11, 12]. Depression is associated with an increased risk of MI, and it may therefore complicate the interpretation of the study results [13–18]. Only one randomized clinical trial comparing cardiovascular event rates in antidepressants and placebo has been published [9]. In that study, a 30 % lower, but nonsignificant, risk of MI was observed in users of sertraline compared with users of placebo treatment. Tricyclic antidepressants (TCAs), which decrease platelet activation and aggregation to some extent [3, 4], were associated with higher risk of MI [10, 11].

Thus, despite evidence of decreased blood platelet aggregation in in vitro experiments with antidepressants, more studies are required to shed light on this association. When we have more insights on the antiplatelet effects of antidepressants in the general population, clinical practice might benefit by adjusting treatment strategies. Studies using time-varying exposure of antidepressants as well as controlling for depression might provide further insights. Within this study, we aimed to investigate the association between use of any antidepressant and risk of MI in the general middle-aged and older population.

## Methods

### Study setting

The current study was conducted in the prospective population-based Rotterdam Study, designed to investigate risk factors for age-related diseases. From 1990 to 1993, all inhabitants aged 55 years and older from the Ommoord district located in Rotterdam, The Netherlands, were invited to participate in RS-I. In total, 7983 individuals agreed to participate (response rate 78 %). In 2000, all inhabitants of Ommoord aged 55 years and older were asked to participate in an extension of the original cohort when they were not previously invited. In total, 3011 individuals agreed to participate (response rate 67 %). Follow-up examinations were conducted every 3–4 years after baseline. A more detailed description of the Rotterdam Study is published elsewhere [19, 20]. The Rotterdam Study has been approved by the medical ethics committee according to the “Wet Bevolkingsonderzoek: ERGO” (Population Study Act Rotterdam Study), executed by the Ministry of Health, Welfare and Sports of The Netherlands, and written informed consent was obtained from all study participants.

### Study population and design

For the present study, we included all participants from the Rotterdam Study cohorts free of MI at baseline. Within the prospective cohort, cases of MI were matched to all eligible participants in the cohort without MI with the same sex, and a similar age (±1 year) at the event date. For every matched set, the exposure status to antidepressants and covariates in each case and its corresponding controls was assessed on the event date as described below. Cases were censored at the event date, whereas controls were allowed to develop MI at a later date during the course of follow-up.

### Antidepressant drug exposure

More than 95 % of the participants have their drug prescriptions filled at one of the seven regional pharmacies. From 1 January 1991 onwards, complete dispensing data is available on a day-to-day basis, which includes the Anatomical Therapeutical Chemical (ATC) code of the drug [21], the dispensing date, the total number of drug units per prescription, the prescribed daily number of units, and the product name of the drug.

A dispensing episode was calculated by dividing the total number of filled tablets/capsules by the daily prescribed number. Antidepressant use (based on ATC code, N06A) was additionally subdivided into TCAs (ATC code = N06AA), SSRIs (ATC code = N06AB), and other antidepressants (ATC code = N06AX). Participants were defined as current user when the event date fell within a dispensing episode. Participants were considered past users, if they previously filled an antidepressant, TCA or SSRI dispensing during follow-up, but were not current users. The period during which participants were not using antidepressants on the index date and had not used them in the past was defined as never use.

### Validation of the study outcomes

Both cohorts were continuously monitored for the occurrence of major morbidity and mortality through linkage with the records from the general practitioner. MI (fatal and nonfatal) was adjudicated based on a combination of symptoms, ECG measurements, and enzyme markers indicative of the presence of MI, as described in more detail elsewhere [22]. All potential MI cases were independently adjudicated by two research physicians. A medical specialist, whose judgment was considered final, reviewed all potential cases.

All-cause mortality was based on information from the Central Register of Population of the municipality of Rotterdam and collaborating general practitioners.

### Covariates

The following covariates were considered as potential confounders: body mass index (BMI), systolic and diastolic blood pressure, highest reached level of education, high-density lipoprotein (HDL) and total cholesterol levels, treated diabetes mellitus, smoking status, history of heart failure, history of venous thromboembolism, presence of depression and/or anxiety, use of blood-pressure-lowering drugs, beta-blockers, cholesterol-lowering agents, anxiolytics, hypnotics, and antipsychotics. A detailed description of how these variables were collected is given in an Online supplementary material.

### Statistical analyses

The association between current antidepressant use and risk of MI was studied using conditional logistic regression analyses with never users of antidepressants as the reference population (analysis 1). This analysis was conducted using unimputed (analysis 1A) data and data after multiple imputations (5 times; 6.5 % maximally missing; analysis 1B). In addition, we also compared the risk of MI between current and past use of antidepressants (analysis 2). We present results for all models unadjusted (model 1) and multivariably adjusted (models 2 and 3). Model 2 was adjusted for all covariates that were considered potential confounding factors. Model 3 is additionally adjusted for covariates that were considered potential intermediate factors, based on previous research [23–26]. The potential intermediate factors were BMI, total cholesterol, HDL cholesterol, use of statins, and diabetes mellitus. The analyses conducted using model 2 were repeated per antidepressant drug class (TCA and SSRI). Because use of other antidepressants was low, we did not evaluate those separately. We additionally conducted analyses in which the period of past antidepressant use started 7, 14, and 30 days after the last day of a filled treatment period to eliminate potential withdrawal effects.

A number of additional analyses were conducted. First, we assessed the association with the number of concomitantly prescribed cardiovascular drugs (ATC codes: “B01A,” “C02,” “C03,” “C07,” “C08,” “C09,” “C10AA”) and studied the percentage of antidepressant use within these strata. In addition, we restricted our population to cases and controls that used at most one of these cardiovascular drugs, and thus excluding those with the highest cardiovascular risk, and repeated the analyses of the risk of MI. And second, we studied the association between current use of antidepressants and mortality (compared with nonuse and past use of antidepressants). The dataset for the analysis on all-cause mortality was similarly constructed as for the analysis on MI, as is described above. Results from these analyses provide arguments whether results in the overall analysis were subjected by residual confounding and/or confounding by indication.

We used IBM SPSS Statistics (version 21.0, IBM Corp., Somers, NY, USA) for all analyses.

## Results

### Characteristics of the study population

A total of 744 MI cases (during 101,664 person years) were successfully matched to controls from the total cohort of 9499 participants (Table 1). At baseline, participants who developed an MI during follow-up were, on average, 69.7 years(standard deviation (SD), 8.1), and 44.8 % were women. The total cohort had a mean age of 69.4 years (SD, 8.7) at baseline, and 60.9 % were women.

**Table 1:** Baseline characteristics of the study population

	Baseline characteristics of future MI cases (*N* = 744)	Baseline characteristics of the total cohort (*N* = 9499)
Age in years (mean (SD))	69.7 (8.1)	69.4 (8.7)
Female (*N* (%))	333 (44.8)	5787 (60.9)
Body mass index in kg/m^2^ (mean (SD))	26.8 (3.4)	26.7 (3.8)
Current smoking (*N* (%))	194 (26.1)	1908 (20.1)
Education (*N* (%))		
Basic	145 (19.5)	1,870 (19.7)
Low	295 (39.5)	4,156 (43,8)
Medium	215 (28.9)	2,465 (26.0)
High	90 (12.1)	1,008 (10.6)
Systolic blood pressure in mmHg (mean (SD))	146 (21)	132 (21)
Diastolic blood pressure in mmHg (mean (SD))	77 (11)	77 (11)
Total cholesterol in mmol/L (mean (SD))	6.7 (1.2)	6.4 (1.2)
HDL cholesterol in mmol/L (mean (SD))	1.2 (0.3)	1.4 (0.4)
History of venous thromboembolism (*N* (%))	2 (0.3)	13 (0.1)
History of heart failure (*N* (%))	22 (3.0)	239 (2.5)
Depression (*N* (%))	4 (0.5)	86 (1.0)
Anxiety (*N* (%))	2 (0.3)	53 (0.6)
Glucose-lowering agents (*N* (%))	73 (9.8)	476 (5.0)
Antithrombotic agents (*N* (%))	86 (11.6)	1035 (10.9)
Blood-pressure-lowering agents (*N* (%))	172 (23.1)	2098 (22.1)
Beta-blockers (*N* (%))	142 (19.1)	1246 (13.1)
Lipid-lowering agents (*N* (%))	40 (5.4)	524 (5.5)
Antipsychotics (*N* (%))	5 (0.7)	87 (0.9)
Anxiolytics (*N* (%))	31 (4.2)	453 (4.8)
Hypnotics (*N* (%))	47 (6.3)	540 (5.7)

Abbreviations: *N* number of participants, *SD* standard deviation, *HDL* high-density lipoprotein

### Antidepressant use and risk of MI

Of the 744 MI cases, 19 were current users and 93 were past users of antidepressants (Table 2). Compared with never use of antidepressants, current use of any antidepressant was associated with a lower risk of MI (analysis 1B, model 1: odds ratio (OR), 0.71; 95 % confidence interval (CI), 0.51–0.98) after adjustment for confounding factors (model 2). These results remained similar when adjusted for potential intermediate factors (model 3). We observed no association between past use of antidepressants and the risk of MI after adjustment for confounding factors (analysis 1B, model 2; OR, 1.17; 95 % CI, 0.95–1.45).

**Table 2:** Association between antidepressant use and myocardial infarction

	Percentage^a^	Events	Model 1^b^ OR (95 % CI)	Model 2^c^ OR (95 % CI)	Model 3^d^ OR (95 % CI)
Analysis 1A^e^					
Never use	84.9	553	1 (reference)	1 (reference)	1 (reference)
Current antidepressant use	3.7	17	0.82 (0.59–1.14)	0.76 (0.54–1.07)	0.76 (0.54–1.07)
Past antidepressant use	11.4	80	1.14 (0.91–1.42)	1.12 (0.90–1.40)	1.12 (0.89–1.40)
Analysis 1B^f^					
Never use	85.0	632	1 (reference)	1 (reference)	1 (reference)
Current antidepressant use	3.8	19	0.77 (0.56–1.06)	0.71 (0.51–0.98)	0.71 (0.51–0.98)
Past antidepressant use	11.2	93	1.18 (0.95–1.46)	1.17 (0.95–1.45)	1.17 (0.94–1.44)
Analysis 2^g^					
Past use	11.2	93	1 (reference)	1 (reference)	1 (reference)
Current antidepressant use	3.8	19	0.72 (0.42–1.23)	0.57 (0.32–0.99)	0.56 (0.32–0.99)

Abbreviations: *95 % CI* 95 % confidence interval, *OR* odds ratio

^a^As we studied the associations with time-varying exposure analysis, controls contributed more than once in the computations before they were censored or became a case. For this reason, exposure is reported as a percentage

^b^Matched on age and sex, further unadjusted

^c^Matched on age and sex, and adjusted for: history of deep venous thrombosis, history of heart failure, systolic and diastolic blood pressure, highest obtained level of education, total cholesterol, high-density lipoprotein cholesterol, smoking, blood-pressure-lowering agents, antithrombotic agents, antipsychotic agents, anxiolytics, hypnotics, depression and anxiety

^d^Model 2 and additionally adjusted for the potential intermediate factors: body mass index, HDL cholesterol, total cholesterol, statins and diabetes mellitus

^e^Analyses with never use of antidepressants as reference, using unimputed data

^f^Analyses with never use of antidepressants as reference, using imputed data

^g^Analyses with past use of antidepressants as reference, using imputed data

With past use of antidepressants as reference, current antidepressant use was associated with a lower risk of MI (model 2; OR, 0.57; 95 % CI, 0.32–0.99), which remained similar when additionally adjusted for potential intermediate factors (model 3), as well as when the period of past use was started later during follow-up (results not shown).

### SSRIs, TCAs, and risk of MI

Compared with never use of SSRIs, current use of SSRIs was associated with a lower risk of MI, although marginally not statistically significant (OR, 0.65; 95 % CI, 0.41–1.02) (Table 3). Past use of SSRIs was associated with a higher risk of MI (OR, 1.42; 95 % CI, 1.06–1.49) compared with never use of SSRIs. A similar point estimate of current SSRI use was observed when compared with past use of SSRIs, although not statistically significant (OR, 0.58; 95 % CI, 0.23–1.49). These results did not materially differ after additional statistical adjustment for potential intermediate factors (results not shown).

**Table 3:** Association between individual antidepressant drug classes and incident myocardial infarction

	Use of SSRIs			Use of TCAs		
	Percentage^a^	Events	OR (95 % CI)	Percentage^a^	Events	OR (95 % CI)
Analysis 1A^b^						
Nonuse	92.2	595	1 (reference)	90.6	587	1 (reference)
Current use	1.8	7	0.70 (0.42–1.17)	1.6	9	0.84 (0.53–1.33)
Past use	6.0	48	1.33 (0.97–1.83)	7.8	54	1.05 (0.79–1.41)
Analysis 1B^c^						
Nonuse	92.4	681	1 (reference)	90.7	672	1 (reference)
Current use	1.8	8	0.65 (0.41–1.02)	1.6	10	0.80 (0.52–1.24)
Past use	5.8	55	1.42 (1.06–1.90)	7.7	62	1.04 (0.79–1.38)
Analysis 2^d^						
Past use	5.8	50	1 (reference)	7.7	54	1 (reference)
Current use	1.8	7	0.58 (0.23–1.49)	1.6	10	0.60 (0.26–1.41)

Matched on age and sex and adjusted for: history of deep venous thrombosis, history of heart failure, systolic and diastolic blood pressure, maximum level of education, total cholesterol, smoking, blood-pressure-lowering agents, antithrombotic agents, antipsychotic agents, anxiolytics, hypnotics, depression and anxiety, and current use of the other antidepressant drug classes (including other antidepressants)

Abbreviations: *95 % CI* 95 % confidence interval, *OR* odds ratio, *SSRIs* selective serotonin reuptake inhibitors, *TCAs* tricyclic antidepressants

^a^As we studied the associations with time-varying exposure analysis, controls contributed more than once in the computation of the odds ratios before they were censored of became a case. For this reason, exposure is reported as a percentage

^b^Analyses with never use of antidepressants as reference, using unimputed data

^c^Analyses with never use of antidepressants as reference, using imputed data

^d^Analyses with past use of antidepressants as reference, using imputed data. For the analysis on SSRIs, 1 case currently using had no matched controls and 5 cases who were past user had no matched controls. For the analysis on TCAs, 8 past users could not be matched. These were therefore not included in the analyses

Compared with never use, as well as with past use of TCAs, current use of TCAs was not associated with a lower risk or MI (Table 3), although the point estimate of current use compared with past use was lower.

### Additional analyses

**Fig. 1:**
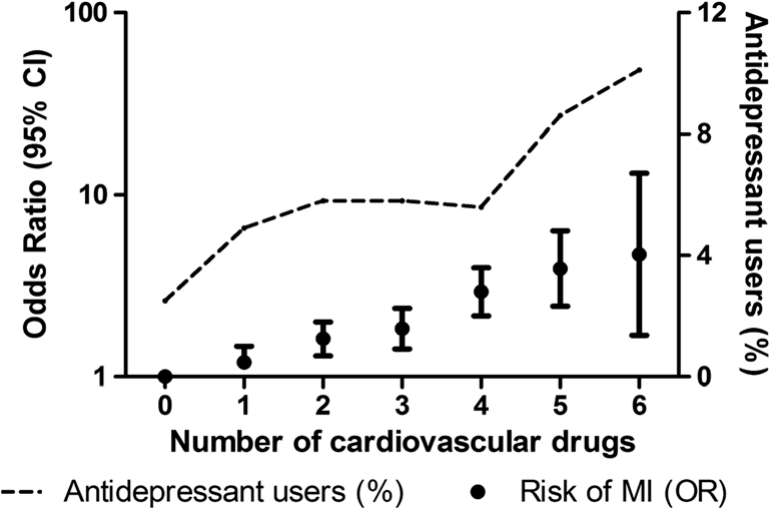
Association between concomitantly used cardiovascular drugs and incident myocardial infarction and the use of antidepressants. Abbreviations: *CI* confidence interval, *MI* myocardial infarction, *OR* odds ratio. Analyses adjusted for age, sex, and body mass index

Participants who used multiple cardiovascular drugs had a higher risk of MI (Fig. 1). Furthermore, they used antidepressants more frequently than participants who used no cardiovascular drug at the index date. After exclusion of participants using multiple cardiovascular drugs at the index date, results for the association between current use of antidepressants and MI remained similar (OR, 0.63; 95 % CI, 0.38–1.06) after adjustment for confounding factors.

Current use of antidepressants was associated with a higher risk of all-cause mortality than nonusers of antidepressants (OR, 1.22; 95 % CI, 1.08–1.38). This was also observed in current users of SSRIs (OR, 1.32; 95 % CI, 1.10–1.58). However, no difference in risk of all-cause mortality was observed between current and past use of any antidepressants, and SSRIs specifically (SSRIs: OR, 0.95; 95 % CI, 0.80–1.12). Furthermore, current and past use of TCAs was not associated with the risk of all-cause mortality.

## Discussion

Within the present case-control study, nested in the prospective Rotterdam Study cohort, current use of antidepressants was associated with a lower risk of MI. Of the different antidepressant drug groups, SSRI use was associated with the lowest risk of MI, although marginally not statistically significant and the number of cases using SSRIs was low.

These findings were supported by some of our extra analyses. First, past use of SSRIs was associated with a nonsignificantly higher risk of MI which suggests that in people with the same indication, discontinuation of SSRIs is followed by disappearance of a protective effect. Because current use of antidepressants was not associated with an increased risk of all-cause mortality (e.g., the increased risk of all-cause mortality was not observed in the comparison between current and past use of antidepressants), the association was not explained by confounding due to competing risk. Second, results remained similar when current use of antidepressants was compared with past use of antidepressants. Past users of antidepressants were assumed to be more comparable with current users of antidepressants with respect to confounding factors than never users. However, the population of past users was considerably smaller than the group of never users. And third, results remained similar after exclusion of participants with multiple concomitantly dispensed cardiovascular drugs, indicative of the participants with the highest cardiovascular risk.

Our findings are also supported by a number of other epidemiological studies. Our observation was similar to a previously published case-control study on the risk of MI in users of SSRIs [5]. Furthermore, a lower risk of MI was observed in users of antidepressants with a high affinity to the serotonin reuptake transporter, and thus may indicate a specific effect of serotonin inhibition [6]. However, none of the other published studies reported a lower risk of MI in users of SSRIs [7–9] or even observed a higher risk [11, 12]. Differences in study design and availability of information on covariates (specifically depression [13–18]) might explain these contradictory results published in the literature on this topic. Furthermore, to the best of our knowledge, no studies have yet been conducted investigating whether the risk of antidepressant-associated bleedings is different between men and women. In previous studies, it has been shown that women have a better response to SSRIs than men [27, 28]; the risk of MI might therefore also be different between men and women. However, in the present study, we had a too low number of cases to study this hypothesis.

In our study population, we did not observe a decreased risk of MI in users of TCAs. Previous studies showed that the use of TCAs was associated with an increased risk of MI [6, 10, 11]. However, when compared with past use of TCAs, a lower risk of MI was observed during TCA use, although not statistically significant due to the low number of cases that used a TCA.

Besides data from some of the conducted epidemiological studies, also data on the relation between serotonin and MI support our findings. The serotonin receptor 2A (5-HT_2A_) and the serotonin transporter are both expressed at the surface of blood platelets and facilitate the activation and aggregation of blood platelets as well as coronary vasoconstriction [29, 30]. In patients with depression it has been shown that sensitivity of the 5-HT_2A_ receptor is increased and expression of the serotonin transporter decreased [4]. This was thought to be one of the explanations why patients with depression are at a greater risk of MI [4]. Also, a high serotonin concentration in serum was associated with a higher risk of MI [31]. Furthermore, use of SSRIs was associated with a decreased serotonin and platelet concentration in whole blood and with a lower platelet activation [32, 33].

For case-control and cohort studies, results might be subject to residual confounding. To our knowledge, only one randomized clinical trial investigating the association between sertraline and risk of MI has been conducted as a secondary study outcome. Within that study, a 30 % lower risk of MI was observed in the sertraline-treated patients after a 24-week treatment period compared with patients on placebo treatment [9]. However, this difference was not statistically significant, probably because of the low number of MI cases. Both groups had a similar relief in depressive symptoms. Thus, a difference in depression during follow-up did not explain the lower risk of MI in the sertraline-treated patients [13–18]. Together with the results of our study, these are additional arguments in favor of a protective effect of antidepressants, in particular SSRIs, on the risk of MI.

Antidepressants, and specifically SSRIs, have been associated with an increased BMI [23], elevated serum low density lipoprotein cholesterol levels [24, 25], and a higher risk of diabetes mellitus [26], which have all been associated with a higher MI risk. However, although additional adjustment for these factors did not materially change the observed associations, it is unlikely that antidepressants decrease the risk of MI more than statins do in the first-line prevention of cardiovascular diseases [34]. In addition, it is worth noting that the use of antidepressants, and especially the SSRIs, has also been associated with an increased risk of bleeding, presumably through the effects of antidepressants on blood platelets [35].

This study has strengths and limitations. First, the available pharmacy dispensing records allowed us to study drug use at the date of MI. We were able to study antidepressant drug use in a time-dependent manner and are able to clearly define episodes during which participants filled antidepressant drug prescriptions. Second, MI adjudication was done using standardized definitions using hospital discharge letters and records of the general practitioner [22]. Next, our study was limited by the small number of participants with an MI who were current users of antidepressants, which also limits the possibility to study the effect of induction and duration. Moreover, we could not ascertain whether the participants were actually taking the drugs or only picked up their dispensing without initiation of treatment. Furthermore, we had no information available about the reason for stopping antidepressant treatment. However, restricting the period of past use did not change the results. Also, due to the observational nature of the data, the results may be subject to residual confounding.

In conclusion, current use of antidepressants was associated with a lower risk of incident MI. Of the antidepressant drug groups, use of SSRIs showed the lowest risk of MI. However, more studies are required to confirm our results.

## Electronic supplementary material

ESM 1 (DOCX 20 kb)
