# Supplementary material for: Use of antidepressants and the risk of myocardial infarction in middle-aged and older adults: a matched case-control study
Source: Eur J Clin Pharmacol. 2015 Nov 7;72:211–8. doi: 10.1007/s00228-015-1972-2 (PMC4713708; doi:10.1007/s00228-015-1972-2)
Supplement: Supplementary file 1 — (DOCX 20 kb) [file 228_2015_1972_MOESM1_ESM.docx]

**Online supplementary materials: “Use of antidepressants and the risk of myocardial infarction in middle-aged and older adults: a matched case-control study**

**R. Noordam *et al***

*Collection of covariates*

For covariates that were assessed during the examination rounds (e.g., BMI and blood pressure), the assessment closest prior to the index date was considered. BMI was calculated by dividing the weight (in kg) by the height (in meters squared). Blood pressure was measured twice, in a sitting position at the upper right arm. The average blood pressure was used in the analyses. Four categories of education were defined (basic = primary education, low = lower vocational, lower and intermediate general, medium = intermediate vocational, higher general, high = higher vocational and university), similar to the UNESCO classification and has previously been described for the Rotterdam Study[[1](#_ENREF_1), [2](#_ENREF_2)]. HDL and total cholesterol were measured in serum using the CHOD-PAP method (Monotest Cholesterol kit, Boehringer Mannheim Diagnostica) and an automated enzymatic procedure (Hitachi analyzer, Roche Diagnostics, Washington DC), depending on the examination round. Because of the two methods, HDL and total cholesterol transformed in round-specific Z-scores. Treated diabetes mellitus was defined as the use of glucose lowering agents (ATC code: A10). The following drugs were considered based on pharmacy records: antithrombotic agents, statins, the number of concomitantly dispensed blood-pressure lowering drugs, antipsychotics, anxiolytics and hypnotics. Current smoking was assessed during the interviews at every examination round. Heart failure was assessed based on typical signs and symptoms confirmed by objective cardiac dysfunction[[3](#_ENREF_3)]. History of venous thromboembolism was defined by diagnoses (ICPC codes: K93 and K94) and notes made by a general practitioner or medical specialist. A specialist was consulted if two independent research physicians did not meet consensus on a potential case of venous thromboembolism. The diagnoses of depression and anxiety were defined on the basis of patient records of the general practitioner, which is described in more detail elsewhere[[4](#_ENREF_4)]. In short, possible cases of depression and anxiety were scanned from the general practitioner’s records. Available information included information of observations by the general practitioner, as well as diagnoses made by the general practitioner and psychiatrist. Two independent research associates validated all potential cases. A psychiatrist was consulted when no consensus was reached on a potential case.

**Reference list**

1 UNESCO (1976) International Standard Classification of Education (ISCED). In: ed.

2 van Rossum C (1999) Socioeconomic Inequalities in Cardiovascular Disease in an Ageing Population, pp177.

3 Leening MJ, Kavousi M, Heeringa J, van Rooij FJ, Verkroost-van Heemst J, Deckers JW, Mattace-Raso FU, Ziere G, Hofman A, Stricker BH, Witteman JC (2012) Methods of data collection and definitions of cardiac outcomes in the Rotterdam Study. Eur J Epidemiol 27 (3): 173-185

4 Luijendijk HJ, van den Berg JF, Dekker MJ, van Tuijl HR, Otte W, Smit F, Hofman A, Stricker BH, Tiemeier H (2008) Incidence and recurrence of late-life depression. Arch Gen Psychiatry 65 (12): 1394-1401
